# Revival of ecological studies during the COVID-19 pandemic

**DOI:** 10.1007/s10654-021-00830-9

**Published:** 2021-12-24

**Authors:** Jonas Björk, Karin Modig, Fredrik Kahn, Anders Ahlbom

**Affiliations:** 1 Division of Occupational and Environmental Medicine, Lund University, Lund, Sweden; 2 Clinical Studies Sweden, Forum South, Skåne University Hospital, Lund, Sweden; 3 Institute of Environmental Medicine, Unit of Epidemiology, Karolinska Institutet, Stockholm, Sweden; 4 Department of Clinical Sciences Lund, Section for Infection Medicine, Skåne University Hospital, Lund University, Lund, Sweden; 5 Center for Work and Environmental Medicine, Stockholm Region, Stockholm, Sweden

The COVID-19 pandemic has brought ecological studies to the fore. In ecological studies, the units of analysis are groups rather than individuals, usually because available data are on the group-level. The purpose of an ecological study can be to learn about exposure effects on individual-level risks or to learn about effects on the group- or population-level. Keeping this distinction in mind is essential to both the researcher and to the reader of a study, but conflation is common. Ecological studies are indispensable to follow time trends and distributions of disease in populations and the surge of these studies during the pandemic is a testament to this. A commentary in last year’s December issue of EJE also stressed that the essence of epidemiology still is comparison of disease rates in cleverly chosen populations [1]. Comparisons in ecological studies generally only provide weak, if any, evidence for causality on the individual level. They are notorious for the so-called ecological fallacy, which may occur when one assumes that relations on the population level also hold on the individual level. However, the scientific quality may vary considerably depending on the study design, setting and choice of comparison groups. The added value of a study also depends on the current state of knowledge. Ecological studies have provided invaluable insights and helped to advance etiological hypotheses. The Seven Countries Study, started by Ancel Keys in the 1950’s, led to novel understanding of the relation between dietary fat and coronary heart disease [2]. Research by McMahon and others on the relation between reproductive history, lactation, and breast cancer risk is a classic example of how hypotheses originating from ecological associations were subsequently tested in individual-level studies [3]. The INTERSALT study is yet another example, where a key strength was that both within-population and cross-population analyses regarding salt intake and blood pressure were undertaken [4].

Studies on exposures that vary in a natural or quasi-experimental manner across geographical areas or over time may approximate the counterfactual and, thus, provide strong evidence for causality even if the analysis is ecological. Well-known examples are studies of health effects following extraordinary events such as the Dutch Famine 1944/1945, the London Smog 1953 and the radiation downfall after the Chernobyl accident 1986. Rollout of vaccination programs may also have quasi-experimental properties, e.g., if vaccination is introduced in one area but not in another or at a known time point allowing for pre–post comparisons [5]. Thus, despite the unpretentious term ecological, such studies have made significant contributions to our knowledge about exposure-disease associations.

In this issue of EJE, Subramanian and Kumar [6] present two ecological analyses on proportion of vaccinated in relation to frequency of COVID-19 cases. The first analysis compares 68 countries around the globe and the second 2 947 counties within the US. We interpret their research question to be how the proportion of vaccinated impacts the incidence of COVID-19, that is, a question on the population-level. The answer to this question is not straightforward as vaccination may impact incidence not only individually among vaccinated but also contextually among both vaccinated and unvaccinated, e.g. through herd immunity effects [7]. Vaccination may also have other indirect behavioral effects if high population uptake leads to abolishment of public measures to reduce disease transmission, or if people’s behavior for other reasons become riskier as uptake increases. Both individual- and population-level effects of vaccination can, thus, be profound. A multilevel approach is generally required in order to disentangle effects at different levels and avoid ecological as well as individualistic fallacies in inference [8]. With only ecological data at hand are we left with the task to estimate the overall population effect on incidence of disease as accurately as possible. This can be done by using carefully planned inclusion criteria for the comparison groups, together with an appropriate adjustment strategy to account for remaining group differences.

Thus, the design and analysis of an ecological study can be far more complicated than they first appear. Subramanian and Kumar do not put much effort in the design of their ecological analysis across countries. The selection of countries from Our World in Data [9] seems quite arbitrary and differences in for example testing volumes, public measures or seasonality in the pandemic are not accounted for. The presented post hoc comparisons between specific countries do not appear well-motivated. If we were to ask specifically, and counterfactually, what the incidence had been in Iceland, if the proportion vaccinated had been lower, then using South Africa or Vietnam as comparison seems misplaced given all other differences between these countries besides vaccination. Taken together, between-country comparisons without more effort in design or analysis therefore become rather uninformative.

**Fig. 1:**
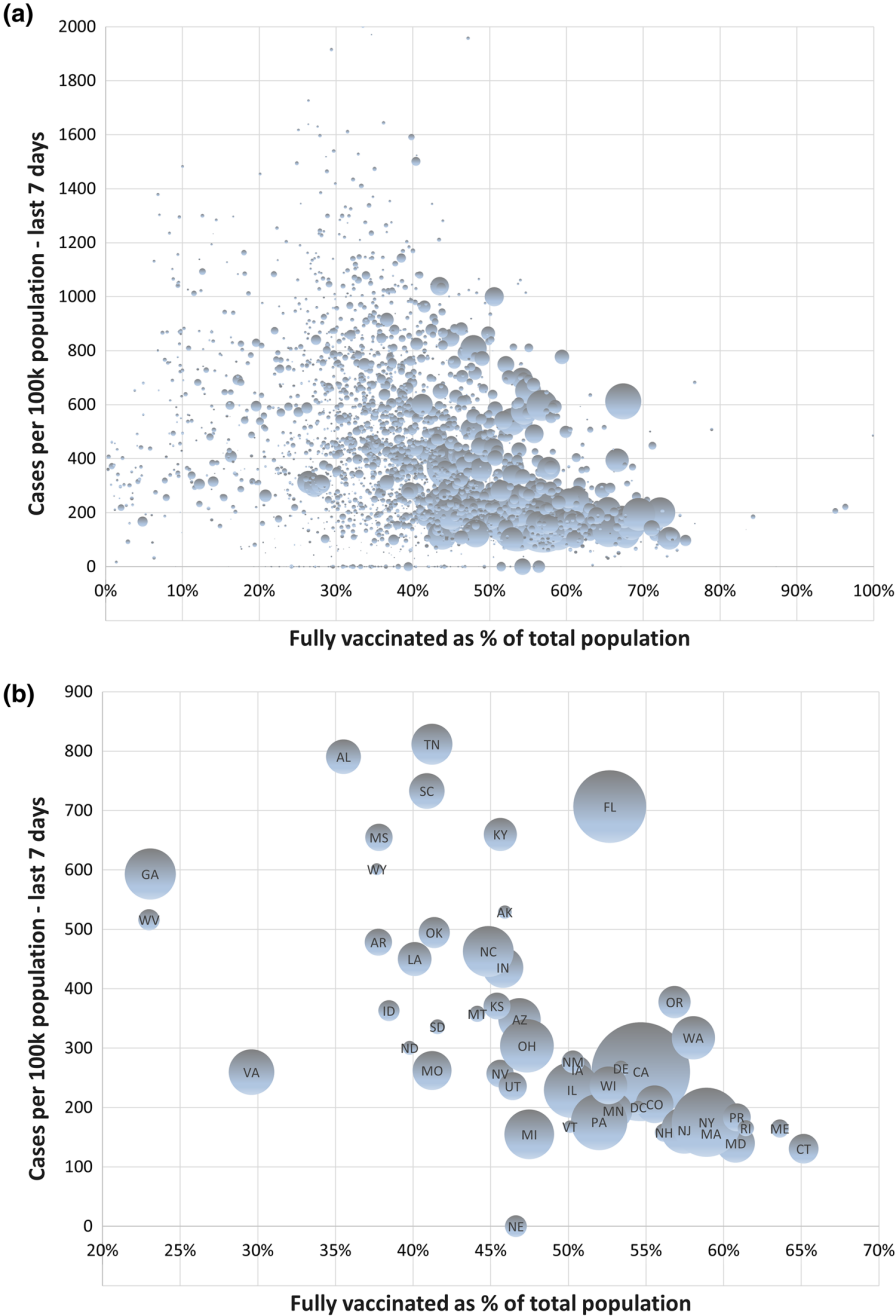
The association between the proportion of fully vaccinated and COVID-19 cases per 100 000 people in the last 7 days as of September 2, 2021 across (**a**) 2947 counties (**b**) 50 states in the US (Texas excluded due to missing data). The area of each bubble is proportional to population size

The comparison of US counties is more promising from a validity perspective, even though important other differences besides vaccination most likely remain also across the counties. The data set available from the White House COVID-19 Team is impressive [10], and offers rich opportunities for in-depth analyses that are left unexploited by Subramanian and Kumar. There is certainly substantial variation in COVID-19 cases across the US counties, but the true heterogeneity in underlying rates is exaggerated unless differences in population size (range 463 to > 10 M across the counties) are taken into account. A bubble graph with each bubble proportional to population size is a simple visual tool that clearly shows that much of the variability in COVID-19 cases occur in counties that are small (Fig. 1A).

By aggregating to the state level, we get a less noisy picture of how large the differences in COVID-19 cases across the US actually were during the study period (Fig.1B).

**Fig. 2:**
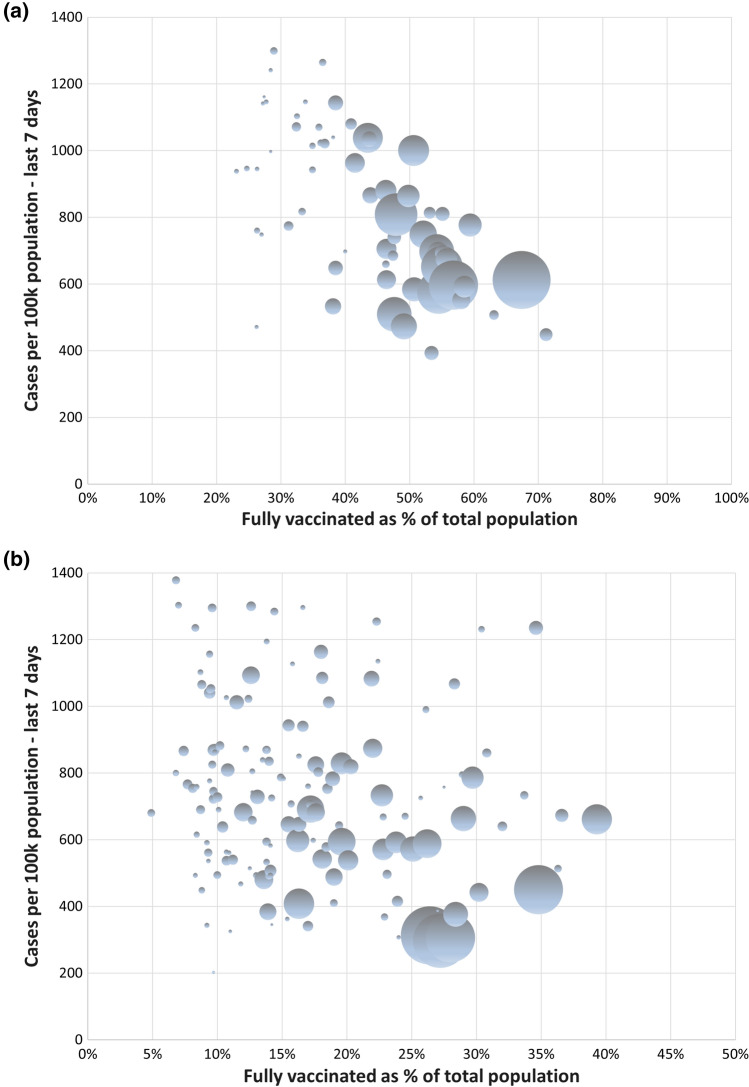
The association between the proportion of fully vaccinated and COVID-19 cases per 100 000 people in the last 7 days as of September 2, 2021 across counties in (**a**) Florida (**b**) Georgia. The area of each bubble is proportional to population size

This graph suggests a clear downward trend with generally fewer COVID-19 cases in states with higher proportions vaccinated. Florida falls out as a clear outlier as a large state with a COVID-19 surge during the study period despite more than 50% fully vaccinated. Arguably, the downside of the aggregation to the state level is that the analysis may be even more prone to ecological fallacy. A natural next step to sharpen the inference from a counterfactual perspective is therefore to zoom into particular states in order to control for some of the differences in vaccination strategies, testing volumes, public restrictions and seasonality. Also, within Florida, COVID-19 cases were inversely related to levels of vaccination (Fig. 2A). Another interesting in-depth analysis can be done for Georgia, the state with the lowest proportion (23%) vaccinated (Fig. 2B). Here too, the overall pattern is that counties with larger proportions of vaccinated generally had fewer cases. As a final analytical step, we would advocate the use of a multilevel model [8], analyzing counties nested within states in order to estimate the overall association between proportion vaccinated and case rate during the study period, potentially with adjustment for aggregate measures of socioeconomic conditions, population density and age structure at the county level.

Thus, a further analysis of the US county data indeed suggests a population-level effect from vaccination on COVID-19 incidence also during the short study period chosen by Subramanian and Kumar. The authors should be complimented for the openness and transparency in the data used, which make additional analyses possible as a basis for scientific discussions of the meaning of their results. The richness of the data [10] facilitates real-time surveillance of overall population effects not only on infections but also on the outcomes that the vaccines are primarily designed to protect against, namely severe disease leading to hospitalizations and deaths.

Despite the simplicity of analysis and presentation, even a carefully designed ecological study cannot provide definite answers to the most urgent issues related to vaccine effectiveness. As individual- and contextual effects cannot be separated, ecological studies are not well suited to inform policy about waning vaccine effectiveness and the appropriate time point for additional booster doses. Furthermore, the difference in protection against infection versus severe disease as well as for how long the effect lasts may also vary depending on type of vaccine. This is something that an ecological study generally cannot address unless different vaccines have been used in separate but comparable geographical areas. Yet, ecological studies are an indispensable part of the toolbox for continuous epidemiological surveillance at the population level. Careful design, analysis and reporting are essential to avoid misinterpretations, not the least by the general public who for long also have been following the COVID-19 surveillance and research in real time.
